# Psychosocial Complications of Coronary Artery Disease

**DOI:** 10.5812/ircmj.18162

**Published:** 2014-06-05

**Authors:** Hossein Karimi-Moonaghi, Mohammad Mojalli, Shahla Khosravan

**Affiliations:** 1 Department of Medical-Surgical Nursing, Faculty of Nursing and Midwifery, Mashhad University of Medical Sciences, Mashhad, IR Iran; 2 Department of Medical education, Faculty of Medicine, Mashhad University of Medical Sciences, Mashhad, IR Iran; 3 PhD Candidate in Nursing, School of Nursing and Midwifery, Mashhad University of Medical Sciences, Mashhad, IR Iran; 4 Social Determents Health Research Center, Gonabad University of Medical Sciences, Gonabad, IR Iran

**Keywords:** Psychology, Coronary Artery Disease, Qualitative Research, Iran

## Abstract

**Background:**

Cardiovascular diseases are the leading causes of death around the world. The coronary artery disease (CAD) is one of the most common diseases in this category, which can be the trigger to various psychosocial complications. We believe that inadequate attention has been paid to this issue.

**Objectives:**

The purpose of the present study was to explore the psychosocial complications of CAD from the Iranian patients’ perspective.

**Patients and Methods:**

A qualitative design based on the content analysis approach was used to collect the data and analyze the perspective of 18 Iranian patients suffered from CAD, chosen by a purposeful sampling strategy. Semi-structured interviews were held in order to collect the data. Sampling was continued until the data saturation. The data were analyzed using qualitative content analysis approach by MAXQUDA 2010 software.

**Results:**

This study revealed the theme of the patients’ challenges with CAD. This theme consisted of: "primary challenges," including doubting early diagnosis and treatment, and feeling being different from others; "psychological issues," including preoccupation, fear of death and surgical intervention, recurrence stress , anxiety and depression; "problems of life," including financial problems, work-related problems, and family-related problems; and "sociocultural issues," including change in perspective of people towards the patient, and cultural issues.

**Conclusions:**

Although the management of physical problems in patients with CAD is important, psychosocial effects of this disease is more important. Thus, health care personnel should pay ample attention to identify and resolve psychosocial problems of these patients. Results of this study can be used to empower these patients.

## 1. Background

Today, because of mechanistic lifestyle, technological progress, overpopulation in urban areas, changes in lifestyle and tendency toward bad habits, cardiovascular diseases (CVD) are increasing worldwide (1). Studies show that cardiovascular diseases are the main cause of death and the third most prevalent disease in Iran. Every day, 378 people die, which 39.3% of deaths are due to CVDs. An important point tragically overlooked globally is that 80% of these deaths can be prevented (2).

Coronary artery disease (CAD) is one of the most important causes of death in the United States (USA). Based on estimates, 1250000 cases of myocardial infarction (MI) happen in the USA every year, which 50000 of them ended in death. The annual global death rate caused by MI is around four million (3). Rate of hospital admission due to CAD has been increased indicating occurrence and recurrence of this disease. Despite hopes for reducing mortality rate, the sharp increase in hospital admission is going to become a growing concern (4).

Coronary artery disease causes various problems for the patient and his/her family. Absence from work, high cost of treatment, and morbidity impose pressure to the community resources. However, CAD prevention can reduce these losses significantly (5).

Patients with the history of MI or coronary artery bypass graft (CABG) surgery experience radical changes in their health status. They should adjust to these changes; otherwise, they will be unable to cope with the health-care plan (6). Although most of the patients recover from a cardiovascular event through a psychosocial approach, at least 25% of them show long-term compliance difficulties. These patients have negative emotional issues such as excitement, anger, anxiety and depression and often fail to return to their previous work, leisure, and level of sexual activity, even if they are physically fit to these activities (7).

In training the medical personnel to deal with MI, the treatment and pathological processes are emphasized more than the psychosocial consequences of the disease. However, long-term quality of life significantly depends on psychosocial outcomes and their management during the medical care (8). Increasing the prevalence of psychosocial complications after CAD and the lack of enough researches on this subject in Iran were the main reasons for us to study the problems of such patients.

## 2. Objectives

This qualitative study aimed to explore CAD psychological complications from the perspective of these patients in Iran to provide the required knowledge for both caregivers and sufferers to prepare an appropriate empowerment process.

## 3. Patients and Methods

### 3.1. Design

We used a qualitative research method with a content analysis approach. Qualitative research is an appropriate tool to get in-depth information from the patient's perspective (9). The research environments were teaching hospitals affiliated to Mashhad and Gonabad Universities of Medical Sciences, Iran. Selection of participants was based on having CAD, including angina pectoris and/or MI and absence of other diseases, particularly mental disorders.

### 3.2. Participants

The participants should express their experiences willingly and coherently. In a qualitative research, the sample size is variable and researchers must continue the sampling process to a point at which a new idea is obtained (10, 11). Thus, data collection was done by purposeful sampling and continued via sampling with maximum variation until data saturation was achieved. Totally, the participants were 18 patients, 12 men and 6 women aged between 45 and 80 years. This research has done in the cities of Gonabad and Mashhad in Iran during 2013 to 2014. Participants were selected by considering the maximum variety in terms of their gender, age, previous experiences in family, type of treatment, duration of disease, educational and job background.

### 3.3. Ethical Issues

At first, we explained the aim of the research to the participants. They were informed that participation in the study was voluntary, and they could withdraw from the study at any time. The participants were reassured that their identity would not be revealed in the research report. Then, the participants signed a written form of consent agreeing the recording of the interview.

This research was approved by the Ethics Committee of the Mashhad University of Medical Sciences (No: 900569).

### 3.4. Data Gathering

Semi-structured interviewing was used for data collection; it is an appropriate method for qualitative researches because of its flexibility and comprehensiveness (10). The principal investigator performed all interviews and transcribed them under supervision of expert supervisors. The interviews lasted between 45 minutes to 1.5 hour with a mean of 1 hour. All the interviews were conducted personally, tape-recorded, transcribed verbatim, reviewed, coded, and then analyzed. In qualitative researches, the researchers must get immersed in the data (10); therefore, they need to listen to the interviews and review the handwritten notes several times.

### 3.5. Data Analysis

Qualitative content analysis was used for subjective interpretation of the text data. In this method, codes and themes are identified via systematic categorization. Content analysis does more than merely extract objective data; it also helps to reveal hidden themes and patterns within the data (12).

 To this end, the analysis was carried out based on Graneheim and Lundman approach (Table 1). The visible and hidden concepts were ascertained, coded, summarized and categorized through the description of the participants, and then themes were extracted from them. The codes were based on meaning units taken from the participants’ descriptions, and were classified according to their similarities and differences (13).

**Table 1.** Steps of Analysis Based on Graneheim and Lundman Approach

Steps	Activity
**Start Point**	The interviews were transcribed
**Step 1**	The texts were read through several times to get a sense of the whole
**Step 2**	Meaning units were extracted from the text
**Step 3**	An abstraction of the meaning units into codes was created
**Step 4**	The various codes were read and re-read and compared against each other. Based on this readings and a reflective process, the codes were sorted out into sub-categories
**Step 5**	The next step in the analysis was to count the occurrence of each sub-category in the interviews
**Step 6**	The sub-categories were compared with each other and with the original text to create mutually exclusive categories
**Step 7**	An independent analysis of all the texts was performed by each of the two co-authors. All authors discussed the categorization and the content and reached a consensus about the categorization

### 3.6. Trustworthiness

Lincoln and Guba (1985) proposed the criteria to enhance the rigor of a qualitative research, which comprised credibility, transferability, dependability and conformability (14). In this study, researchers allocated sufficient time for data collection and established a close communication with participants. The interviews were returned to the participants to verify the accuracy of the results and to validate the congruity of the findings with their experiences as a member checking. The data were coded and categorized by authors. Then emerged themes were compared. Opinions of experts, including three PhD nursing candidates of data analysis, (as peer checking) were asked and discussed for three weeks. Regarding rigor, the research team discussed and interpreted the findings until a consensus was reached. These items enhanced the credibility and dependability of the research.

The principal investigator collected and analyzed the data while the others checked and verified the results. The participants were selected by considering the maximum variety in their gender, age, family history, treatment, duration of disease, educational and job background, to ensure the transferability of the study.

## 4. Results

Our study categorized the theme of patients’ challenges with CAD. This theme consisted of: "primary challenges," including doubting primary diagnosis and treatment, and feeling being different from others; "psychological issues," including preoccupation, fear of death and surgical intervention, stress due to recurrence of disease, anxiety and depression; "problems related to life," including financial problems, work-related problems and family-related problems; and "sociocultural issues," including change in perspective of people towards the patient, and cultural issues. This theme and its subthemes characterize the psychosocial consequences of coronary artery disease in Iranian patients (Table 2).

**Table 2.** Themes, Categories and Subcategories

Subcategories	Categories	Theme
**Doubting primary diagnosis and treatment, Feeling different from others**	Primary Challenges	patients’ challenges with CAD
**Preoccupied with themselves, Fear of death and surgical intervention, Stress of disease recurrence, Anxiety and depression**	Psychological Issues	
**Economic problems, Job-related problems, Family-related problems**	Problems related to Life	
**Change in perspective of people toward patient, Cultural issues**	Sociocultural issues	

### 4.1. Patients’ Primary Challenges

#### 4.1.1. Doubting Primary Diagnosis and Treatment

At the early stages of the disease, participants doubted the primary diagnosis and treatment. Thus, they kept searching for more reliable diagnoses and treatments. They were referred to equipped centers and more-experienced physicians and specialists for consultation. One participant (No. 8) commented: “Patients should consult with expert physicians not just anyone. A doctor, who suggests doing something and going somewhere specific, should be an expert in that field.” 

#### 4.1.2. Feeling of Being Different From Others

Participants felt overprotected by their family and relatives. One participant (No.16) said: “My brothers, children, bride, and groom tell me ‘don’t work’, ‘be careful’, ‘don’t put pressure on yourself’.”

### 4.2. Psychological Issues

#### 4.2.1. Preoccupation

Patients’ thoughts were preoccupied by their disease and its outcome: “What will happen to me? Is it necessary to have a surgical operation? and what are the consequences?” They related almost everything to their disease even if it did not have the slightest relevance to their disease. One participant (No. 2) stated: “I didn’t think about the disease in the past but now if I want to do any work, I feel that this heart disease is with me. It comes to my mind. If I want to do anything this disease is with me.”

#### 4.2.2. Fear of Death and Surgical Intervention

Most of the people suppose that heart disease equals death and because of this, they are terrified even by a chest pain, thinking that they may die from it. One participant (No. 12) said: “From the day I was told that my heart had a problem, I felt anxiety. This is because, I had a peer who was told the same; he paid no heed and died”.

More invasive treatments such as angioplasty, stent placement, and CABG are horrifying for them. One participant (No. 5) commented: “I was terrified when they told me that I must have a heart operation. I told them no, I wouldn’t allow that. They told me that you must have an emergency operation. I was very terrified”.

#### 4.2.3. Stress Due to Recurrence

Recurrence of the disease manifests itself as a persistent worry. One participant (No. 11) pointed out: “Stress doesn’t vanish. This is one problem. Stress isn’t just a word to use and then finished! In fact, it is with me everywhere. It's really disturbing because I can't even go about my daily decision-making without feeling stressed”.

#### 4.2.4. Anxiety and Depression

One participant (No. 10) about anxiety commented: “If the meal was harmful, I thought it was related to this disease, and my heart would hurt. I was worry. My mobile was with me everywhere, and I felt anxiety every day”. Another participant (No. 4) about depression stated: “When someone comes to visit me, they love to see me in the same state as I was before. They expect me to be as I was in the past. Neighbors and relatives got upset, and they tell me that I am depressed because I don’t speak as much as I did”.

### 4.3. Life Problems

#### 4.3.1. Financial Problems

The cost of diagnosis and treatment is very high, especially if the patients need operations such as, angiography, angioplasty, stent placement, or CABG. The problem got worse, if they did not have any kind of complementary health insurance. One of them (No. 18) commented: “It would have been better if I hadn’t had any pressure in my life. It will be a real problem for me if I want to borrow money for hospitalization. This stress affects my disease”.

#### 4.3.2. Work-Related Problems

These patients mostly cannot do their job, especially hard work such as agriculture. Moreover, they also try to be cautious. One patient (No. 9) mentioned: “I can't work in the farm. I can't do any of the things I used to do before my heart attack and surgery. I can't do anything.” 

#### 4.3.3. Family-related problems

After CAD, not only the patients but also their families got worried. However, the family forbids them and tries to do those duties instead. Moreover, going to hospitals and visiting doctors can affect the everyday duties or routine of the patients and their families; therefore, their lives do not follow a natural course either. Another example of this subtheme includes limitations of the patients and their families’ social life. They are unable to go on vacations and journeys or simply visit other people. One participant (No. 6) commented: “Warm weather is harmful for those who have heart disease. They are depriving of facilities. They can’t travel”. Another participant (No. 7) stated: “My family is sensitive about me. They tell me ‘don’t work’, ‘don’t run’, ‘don’t pressure yourself’, ‘don’t go riding’, ‘don’t do any kind of heavy work’ ”.

### 4.4. Sociocultural Issues

#### 4.4.1. Change Perspective of People Toward Patients

People show a change in their views through certain conduct and actions, and this makes the patients lose confidence in themselves and start to isolate from the community. One participant (No. 13) commented: “Others see us vulnerable. They feel that we are not able to do work as we used to. They think we have a disease and should be helped”.

#### 4.4.2. Cultural Issues

Iranian patients try to show that they are humble. For example, they eat the same food as the others in parties and family gatherings. Sweet and fatty foods are usually served in these events which are not healthy for CAD patients. One participant (No. 7) said: “Everyone is used to serve a special, culture-based food at home. We used sweet and fat in our meals in the past. This is our culture. In a party, we usually eat fat, sweet, and the best fruits like the others”.

## 5. Discussion

Findings of this study can assist to understand better the psychosocial complications of CAD in the Iranian community. The subtheme “doubting initial diagnosis and treatment” is consistent with a research done by Afraciabi Far et al. (15) . They believed that making a decision to begin treatment of CAD (as a complicated process) is based on the patient’s feeling regarding the ability to control the signs of the disease. Another subtheme “feeling of being different from others” was also reported in a related research done by Condon et al. emphasizing overprotection as a source of annoyance and not as a boon to the participants (16).

Moeini et al. mentioned that ischemic heart disease has multidimensional effects on patient’s life; there are frequent courses of pain that affect patient’s thoughts and feelings (17). This finding is consistent with the preoccupation theme in our study.

Indratula et al. defined “fear of death and surgical interventions” like our study, and reported that these patients had lost confidence in life and started exhibiting fear of death and disability. Patients were also anxious about the risk of death and inability both before and after their surgery (18).

Arnold et al. highlighted stress of recurrence of the disease and reported medium to high levels of stress after MI even after long term adaptation (19). The existence of depression and anxiety was confirmed by Huffman, who reported it in a high rate in patients with acute coronary syndrome, which affected the long-term outcome of the heart disease (20).

Shah et al. like our study, found the frequent presence of financial problems in the patients suffering from CAD that was a risk-factor worsening the outcome of MI (21). Likewise, Rahimi et al. found financial barriers in health care services and drug therapy concurrent with worsening MI, increasing angina, lowering quality of life, and increasing readmissions (22). Brink et al. showed how a decrease in body health and quality of life after MI had a negative impact on return to work (23). This supports our findings about work-related problems.

Similar to our findings, Svedlund et al. (24), Nasiri et al. (25), and Pashaee et al. (26) paid attention to family-related problems and found many changes in different aspects of life in patients with CAD, including working at home, social life, entertainments, and passing down vacation. These factors can lead to psychosomatic reactions in families. In the field of change in perspective of others toward patients, Linden et al. (27) and Khayyam-Nekouei et al. (28) suggested that there was an immediate necessity for psychosocial interventions in these patients.

With reference to cultural issues, Astin et al. concluded that there may be cultural and ethnical differences in patients and families, which could adversely affect recovery; therefore, health care personnel should bear this in mind when giving service and think up appropriate solutions based on a cultural approach to overcome any problem arising from these differences (29).

Since the heart is one of the most important organs of the body, any heart disorder can be a direct threat to the patient’s identity; thus, the psychosocial complications can be more important than the physical ones. As patients worry about correct diagnosis and treatment of CAD, it is vital that health care personnel pay ample attention to this matter. Sufficient attention must be paid to the psychological and spiritual problems of the patients and giving consultation to alleviate them is strongly suggested.

Furthermore, it is also essential for the mass media to educate the public on how to treat patients with CAD. Adequate focus on financial, work, and family problems is of great importance, too. Last but not the least, physicians and health care personnel must consider patient’s cultural background and traditions in order to overcome any cultural obstacles hindering the process of recovery.

Since the present research studied a limited number of patients, its generalization to other conditions and situations must be performed with caution. We recommend that similar researches conducted on more patients and in other countries.
